# Reliability of Different Mark-Recapture Methods for Population Size Estimation Tested against Reference Population Sizes Constructed from Field Data

**DOI:** 10.1371/journal.pone.0098840

**Published:** 2014-06-04

**Authors:** Annegret Grimm, Bernd Gruber, Klaus Henle

**Affiliations:** 1 Department of Conservation Biology, UFZ – Helmholtz Centre for Environmental Research, Leipzig, Germany; 2 Institute for Biology, Faculty of Biosciences, Pharmacy and Psychology, University of Leipzig, Leipzig, Germany; 3 Institute for Applied Ecology, Faculty of Applied Sciences, University of Canberra, Australian Capital Territory, Canberra, Australia; University of Western Ontario, Canada

## Abstract

Reliable estimates of population size are fundamental in many ecological studies and biodiversity conservation. Selecting appropriate methods to estimate abundance is often very difficult, especially if data are scarce. Most studies concerning the reliability of different estimators used simulation data based on assumptions about capture variability that do not necessarily reflect conditions in natural populations. Here, we used data from an intensively studied closed population of the arboreal gecko *Gehyra variegata* to construct reference population sizes for assessing twelve different population size estimators in terms of bias, precision, accuracy, and their 95%-confidence intervals. Two of the reference populations reflect natural biological entities, whereas the other reference populations reflect artificial subsets of the population. Since individual heterogeneity was assumed, we tested modifications of the Lincoln-Petersen estimator, a set of models in programs MARK and CARE-2, and a truncated geometric distribution. Ranking of methods was similar across criteria. Models accounting for individual heterogeneity performed best in all assessment criteria. For populations from heterogeneous habitats without obvious covariates explaining individual heterogeneity, we recommend using the moment estimator or the interpolated jackknife estimator (both implemented in CAPTURE/MARK). If data for capture frequencies are substantial, we recommend the sample coverage or the estimating equation (both models implemented in CARE-2). Depending on the distribution of catchabilities, our proposed multiple Lincoln-Petersen and a truncated geometric distribution obtained comparably good results. The former usually resulted in a minimum population size and the latter can be recommended when there is a long tail of low capture probabilities. Models with covariates and mixture models performed poorly. Our approach identified suitable methods and extended options to evaluate the performance of mark-recapture population size estimators under field conditions, which is essential for selecting an appropriate method and obtaining reliable results in ecology and conservation biology, and thus for sound management.

## Introduction

Knowledge of population size is of key importance in many fields of animal ecology, evolution, and conservation biology. For natural populations of animals, it is rarely possible to count all individuals. Thus, usually estimation methods have to be used. Capture-mark-recapture (CMR) is a commonly used approach for estimating population size [1], [2], [3], [4], [5]. Meanwhile, a huge range of different statistical models exists for analysing CMR data [5], [6], [7], [8]. Field biologists are faced with the difficulty of deciding which approach to use and how reliable the selected method is for the populations they study [5], [6], [8]. This problem is exacerbated by the existence of a range of alternative methods using similar biological assumptions about the capture process. Consequently, good recommendations based on field data for the most suitable methods for various natural populations are needed to validate and complement simulation studies.

The performance of CMR models depends on their assumptions, how these assumptions can be met in the field, and on the robustness of the estimators to violations of the underlying assumptions. Critical assumptions are whether capture probability remains constant, changes with time or as behavioural response to previous experience, or varies among individuals [2], [4], [7]. Because population size must be known to assess the performance of estimators, assessments usually rely on virtual CMR studies that create capture histories under different assumptions about capture probabilities [9], [10], [11]. The advantage of such simulation studies is that they allow assessment of the performance of estimators by systematically varying capture probability.

An important limitation of simulation studies is that it is unclear to which extent the variation in capture probability implemented reflects the variation occurring in nature [11], [12]. Thus, it is important to study the performance of various estimators under field conditions. For this purpose, it is essential to have populations of known size available. As this is rarely the case, few such studies exist and most compared only a small number of methods. These studies either used penned populations of known size [12], [13], [14], [15], [16], [17] or compared estimates to the number of individuals obtained in complete removals from areas of limited size, e.g., small pools [18], ant nests [19], or fenced-off areas [20].

Intensively studied closed populations and the use of a subset of the data to estimate population size may offer an additional opportunity that seems not to have been used so far. Here we explore this approach using a very intensively studied closed population of the Australian gecko *Gehyra variegata* [21]. We assessed the performance of ten different methods without covariates and two different sets of methods including covariates. As behavioural observations suggested that individual heterogeneity may be present [22], we focused on methods that allow individual heterogeneity. We evaluated a set of models in programs MARK [23], [24], the most widely used tool to estimate population size, and CARE-2 [25] that allow, in addition to individual heterogeneity, temporal and behavioural change of capture probability. We also assessed a truncated geometric distribution [3] as this distribution has been used in earlier studies to estimate population size of our model species [21]. We further included three modifications of the Lincoln-Petersen estimator since this is a simple, still frequently used method. We predicted that models incorporating individual heterogeneity would perform better than other models studied and that models using covariates or mixture approaches would outperform models that account for heterogeneity in a more simple way.

## Materials and Methods

### Data collection

This research was carried out under permit number A478 NSW National Parks and Wildlife Service. This licence covered all animal ethics considerations as well as a permit to capture and mark the animals.

Mark-recapture data collected from a population of the arboreal, nocturnal gecko *Gehyra variegata* (Duméril & Bibron, 1836) living at the huts of the station in Kinchega National Park (32°28′ S, 142°20′ E), western New South Wales, Australia, provided the basis for the evaluation of the selected population size estimators [21].

The study site included seven huts where geckos were caught by hand at night, measured, sexed, and marked by toe-clipping and with a dorsal colour mark for short-term identification. Toe-clipping had no influence on survival (Höhn et al., accepted). Data collection followed a robust design [26]. The population was sampled intensively bimonthly (primary periods) for two years from September 1985 to March 1987 except July 1986 due to the inactivity of the species. Each primary period consisted of five to sixteen secondary periods (usually consecutive nights).

Potential habitat within a strip of 50 m around the huts was surveyed to detect dispersing individuals [21]. In parallel, a second population living in riverine woodland in a distance of approximately 30 m from the huts was studied and provided additional opportunity to discover dispersing individuals. Over the whole time span, only one subadult gecko moved from the closest tree into the study area and back again within a two month period, implying that there was negligible emigration and immigration, allowing construction of reference population sizes. This conclusion is further corroborated by movement studies in the second population that showed that longer distance movement is very rare [21], [27].

### Construction of reference population sizes

We used two approaches to create reference population sizes assessing whether the relative performance of the evaluated methods remains consistent. In both approaches we determined a reference population for each but the last primary period. In the first approach based on partly independent data, we counted all individuals marked throughout the study period. We then excluded all individuals only captured in previous primary periods. We further excluded juveniles born in later primary periods (as they were not yet part of the population). Juveniles can be identified reliably by size during the first two years after birth [21]. These reference populations are only partly independent from the data used for estimating population size because some animals were only present in the primary period used for analyses (i.e. for these animals the same capture was used to include them in the reference population and to estimate population size). In a second approach, we created a fully independent reference population by excluding additionally all animals captured in, but not after the period analysed. Consequently, we also excluded these individuals from the capture data used for population estimation. By the exclusion of these individuals no capture was used both for constructing the reference population and to estimate the reference population.

Because of the high capture intensity few, if any, individuals should have been missed in creating the reference populations for the first 1–2 primary period(s). Thus they represent the biologically relevant entire number of individuals present that have non-zero capture probability (partially independent data set) respectively the part of the population that survived at least to the next primary period (fully independent data set). Reference populations for later primary periods will increasingly ignore individuals with very low capture probability, which are known to create enormous challenges for capture-recapture analysis [28]. We used these reference populations reflecting artificial subsets of the population to assess whether the relative performance of the tested methods change when few individuals with low capture probability are present. They thus need to be understood as biological entities that provide an alternative way of constructing distributions of capture probabilities that may be generalized in future simulation studies.

To assess whether we may have missed individuals with very low capture probability in our reference populations, we calculated a threshold for daily capture probability (*p_tr_*) above which the expected level of inclusion was at least 95% of all individuals:
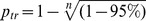
(1)with *n* being the number of capture occasions used to determine reference population sizes.

### Assessment of population size estimators

We used data from November 1985, 1986, January 1986, 1987, and March 1986, 1987 for estimating population sizes since geckos were most active during these months [21]. Minimizing variation of capture probability over time, we combined occasions with very low sampling rates [4].

Our evaluation of estimator performance focussed on models that account for individual heterogeneity since from our experience in the field we expected substantial individual heterogeneity due to different catchabilities among individuals. To mathematically assess whether individual heterogeneity was considerable, we calculated a coefficient of variation (CV) in capture probabilities as suggested by Chao et al. (1992) and Lee and Chao (1994) using program CARE-2 [25], [29], [30]. The CV is a nonnegative parameter that indicates individual heterogeneity, which is larger for higher degrees of heterogeneity among individuals. If and only if individuals are equally catchable, the CV is zero. This heterogeneity is relevant for some of the coverage estimators evaluated and also to understand the different performance of the evaluated estimators.

In total, we assessed twelve estimators. Table 1 provides an overview of the estimators, their characteristics, and where relevant methods select among alternatives within a specific estimation approach. We did not include the spatially explicit capture-recapture (SECR) method [31], [32] although this method reduces individual heterogeneity at spatial level as there is a complex unknown relationship between distances and capture probabilities among individuals making this method not applicable to our data. The first three estimators assessed are from a set of models implemented in programs CAPTURE and MARK [2.24]. The models in CAPTURE make complementary assumptions about capture probability. Capture probability may be constant (M_0_), variable in time (M_t_), among individuals (M_h_), or due to trap shyness or trap happiness (M_b_), and all pairwise combinations thereof. There is no estimator for the most general model, M_tbh_. Model selection is made by a discriminant function that builds on several specific model tests [2]. Besides the estimator chosen by the discriminant function [Appropriate], we evaluated the two M_h_ models implemented in CAPTURE: the interpolated jackknife estimator [IntJK] [33], [34] and the moment estimator [ME] of Chao (1987, 1988), which sometimes is also referred to as the lower bound estimator[35], [36]. Both estimators use capture frequencies to estimate population size. Whereas the nonparametric jackknife estimator is based on linear combinations of all capture frequencies [33], Chao's moment estimator is exclusively based on *f_1_* and *f_2_*, which are the number of individuals captured once or twice [35], [36], [37].

**Table 1:** Overview on all tested population size estimators including their references, basics, and model selection procedures.

Estimator	Reference	Basics	Model selection
Linconln-Petersen (LP)	[3], [4]	Lincoln-Petersen corrected by Chapman	no model selection
Multiple Lincoln-Petersen (MLP)	[3] and recent study	repeated Lincoln-Petersen estimator	no model selection
Mean Petersen Estimate (MPE)	[3]	mean Petersen estimate for each sampling stage	no model selection
MARK Appropriate	[2]	running all models	discriminant function building on several specific model tests
MARK M_h_ Interpolated Jackknife (IntJK)	[31], [32]	linear combinations of all capture frequencies	no model selection
MARK M_h_ Moment Estimator (ME)	[33], [34], [35]	capture frequencies of individuals captured once (*f_1_*) or twice (*f_2_*)	no model selection
CARE M_h_ Sample Coverage 1 (SC1)	[29]	overall proportion of individual capture probabilities and degree of individual heterogeneity	no model selection
CARE M_h_ Sample Coverage 2 (SC2)	[29]	bias-corrected form of SC1	no model selection
CARE M_h_ Estimating Equation (EE)	[11]	behavioural response, individual heterogeneity, and temporal changes as parameters to model capture probabilities	no model selection
Truncated geometric distribution	[3]	fitting capture frequencies to a geometric distribution	no model selection
Finite mixtures	[28]	models individual differences in capture probabilities using a flexible beta-distribution	AIC
CARE/GSRUN	[25]	conditional likelihood approach using the Horvitz-Thompson population size estimator	AIC

We further evaluated the first [SC1] and the second sample coverage estimator [SC2] of Lee and Chao (1994) [29], the estimating equation [EE] of Chao et al. (2001) [11], as well as a set of models that allow inclusion of covariates (sub-program GSRUN) as implemented in program CARE-2 [CARE/GSRUN] [25]. The sample coverage estimator is a nonparametric estimation technique that builds on the proportion of individual capture probabilities included in the data by the animals captured. The population size estimation is further based on an estimation of the degree of individual heterogeneity, i.e. the coefficient of variation of individual capture probabilities [29], [30].

The estimating equation developed by Chao et al. (2001) uses behavioural response, individual heterogeneity, and temporal changes as parameters to model capture probabilities. Hence, calculating population size for different combinations of model assumptions is possible by using only one formula. Currently, no selection process among alternative models is available [5], [11], so we evaluated model M_h_. This estimator can also be seen as an extension to the sample coverage estimator. The calculation of the other set of models (GSRUN) is based on a conditional likelihood approach [38], [39] using the Horvitz-Thompson population size estimator [40]. For that estimator, we used the following covariates: age (juveniles, subadults, adults) and five different types of huts identified according to similar structures, which may result in similar capture probabilities. The model with the lowest AIC was chosen [41].

Moreover, we tested Pledger's (2000) finite mixture model [Finite mixtures][42], which is also implemented in program MARK [24], [43]. The approach models individual differences in capture probabilities using a flexible beta-distribution. The general model is denoted as *π(.)p(t)c(t)N(.)*, with *π* being the probability that an individual belongs to mixture A, *p* is capture probability for the first capture and *c* for the following ones, thus allowing for trap response, *t* signifies that the variable is time specific, and *N* is population size [43]. We used AIC values for model selection [44].

The final four models assessed are the truncated geometric distribution [Tr. geometric distribution] [3] and three versions of the Lincoln-Petersen estimate. In the first approach, population size is estimated by fitting capture frequencies to a truncated geometric distribution [3]. We wrote an R [45] package for this purpose which was submitted to CRAN [46]. This estimator was tested as it has been used frequently in the past, including for our data set [1], [14], [21], [47], [48].

Lincoln-Petersen estimators are known to be very vulnerable to deviations from equal catchability, but since they are easy to calculate and therefore often used, we included them in these comparisons. We calculated the Lincoln-Petersen estimator [LP] with the adjustments suggested by Chapman [3], [4]. For an odd number of occasions, we split the data in such a way that the difference in number of captures between the two samples was minimized [9]. We also evaluated the mean Petersen estimate [MPE] [3], which is the mean of the Petersen estimates calculated for each stage of sampling, with the number of marked individuals in the population based on the combined data of all previous sampling occasions of the primary period. This approach results in *k*-1 estimates (with *k* denoting the number of trapping samples). Ignoring covariances, as they should be low compared to variances, the variance of the MPE is the sum of all single variances divided by *(k-1)^2^* [3]. As an alternative version, we invented a new way to estimate population size using repeated Lincoln-Petersen estimators, which we call multiple Lincoln-Petersen [MLP]. We took the average of *k-1* Lincoln-Peterson population size estimates that were calculated by pooling the data as follows: for the first case, we used data from the first occasion as *n_1_* and pooled all remaining occasions for *n_2_*; we then pooled occasions one and two for *n_1_* and the remaining occasions (three to *k*) for *n_2_* and so on. We calculated the variance of this multiple Lincoln-Petersen estimate in the same way as suggested by Seber (1982) for MPE. By combining the data from several occasions, all capture probabilities will be increased and the range of capture probabilities will be reduced, thus also reducing heterogeneity. Both should result in improved estimates. Furthermore, this approach should correct for time effects as it uses different combinations of the occasions. In contrast to MPE, this effect applies also to *n_2_*; thus we expected that it should improve the performance of the Lincoln-Peterson approach.

### Estimator ranking

We compared the performance of the estimators based on the coverage of the reference population sizes by their 95%-confidence intervals (CI), the mean span of these confidence intervals, either as provided by the programs or calculated from the variance of the estimated population size. We further ranked them based on the mean of their bias, precision, and accuracy [49] across the reference populations: 
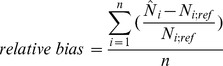
(2)

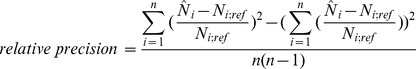
(3)

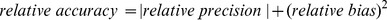
(4)with 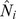 being the estimated population size, *N_i;ref_* the reference population size, and *n* the number of reference populations used to evaluate the estimators.

Relative bias measures the divergence from the reference population size, and relative precision (or relative variance) can be interpreted as the variation in estimates of the reference population size. Relative accuracy combines both measures and can be interpreted as mean square error. These values were computed over all primary periods except the last one (as there was no reference population size).

We ranked all estimation methods using all four criteria, whereby the closer a value is to zero the better is the performance of the estimator. These rankings were done in R [45].

## Results

### Partly independent data sets

**Figure 1:**
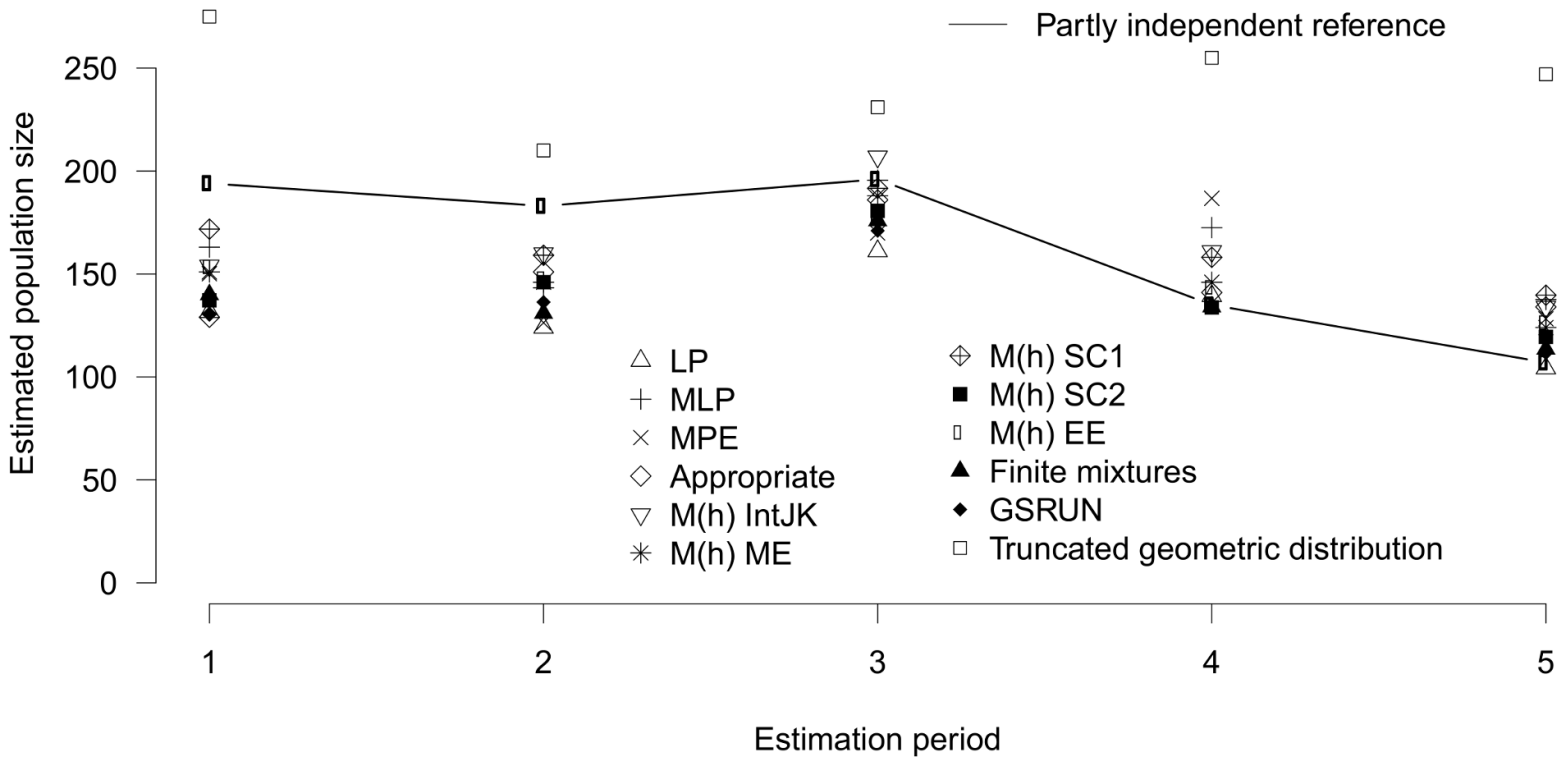
Population size estimates of partly independent entities. Comparison of different methods for population size estimates with the partly independent reference population sizes (connected by a line). LP: Lincoln-Petersen; MLP: Multiple Lincoln-Petersen; MPE: Mean Petersen estimate; IntJK: Interpolated jackknife; ME: Moment estimator; SC1: Sample coverage 1; SC2: Sample coverage 2; EE: Estimating equation.

For the partly independent data sets, all estimators, except the truncated geometric distribution, underestimated the reference population size in the first two primary periods (Fig. 1). In the first period, the SC1 estimate was closest to the reference population size but still substantially biased; in the second period, bias was limited for the SC1 and the IntJack estimators. All methods overestimated the reference population size in the last primary periods, except the Lincoln-Petersen estimators. For the remaining two primary periods, most methods resulted in estimates close to the reference population size. Capture intensity was high for the first three primary periods so that very few, if any, individuals may have been missed in our reference populations, even if individual capture probability was as low as 0.078 (Table 2). The last primary period, in contrast, probably did not include all individuals with low capture probability since daily threshold capture probability for which 95% of individuals are expected to be included in the reference population was 0.259. The coefficient of variation among individual capture probabilities was found to be around 0.6 except for primary periods in November 1985 (0.43) and November 1986 (0.33).

**Table 2:** Results for population size estimation with partly independent data.

Sample period	1985_11	1986_01	1986_03	1986_11	1987_01
Reference	194	183	196	135	107
f_1_	53	50	56	54	43
f_2_	19	26	31	31	23
f_3_	4	14	24	12	10
f_4_	1	4	15	2	5
f_5_	0	1	6	0	3
f_6_	0	3	3	0	0
f_7_	0	0	2	0	0
f_8_	0	0	0	0	0
S	77	98	137	99	84
p_tr_	0.078	0.092	0.122	0.171	0.259
CV	0.43	0.59	0.60	0.33	0.62
LP	131.59 (92.29–170.89)	123.97 (105.86–142.09)	161.1 (145.54–176.66)	**139.34 (112.42–166.27)**	**104.21 (88.71–119.71)**
MLP	**163.04 (131.92–194.16)**	143.30 (132.82–153.78)	**194.56 (185.55–203.57)**	172.53 (155.89–189.18)	137.59 (126.59–148.59)
MPE	150.13 (109.95–190.30)	126.33 (111.02–141.63)	169.90 (158.49–181.31)	**186.73 (129.56–243.89)**	128.23 (112.51**–**143.95)
MARK Appropriate	129 (106–170)	**151 (126–199)**	**186 (165–224)**	**141 (119–186)**	134 (114–170)
MARK M_h_ IntJK	**154 (132–213)**	**160 (134–204)**	**207 (178–256)**	161 (137–200)	134 (114–170)
MARK M_h_ ME	**151 (114–227)**	**146 (122–195)**	**188 (163–235)**	**146 (123–191)**	**124 (103–169)**
CARE M_h_ SC1	**171.8 (126.87–257.32)**	**159.1 (131.96–200.21)**	**191.6 (169.59–220.55)**	**158.1 (132.70–197.78)**	139.7 (114.37–178.96)
CARE M_h_ SC2	**137.2 (110.36–206.13)**	146.0 (122.22–181.47)	**180.7 (161.17–208.29)**	**133.8 (117.95–164.21)**	**119.5 (99.79–154.64)**
CARE M_h_ EE	**153.4 (116.21–221.12)**	147.6 (125.62–177.25)	**177.9 (161.07–199.69)**	**143.5 (124.11–173.4)**	**126.3 (107.53–155.95)**
Tr. geometric distribution	275 (207–378)	**210 (181–248)**	231 (212–254)	255 (212–313)	247 (207–301)
Finite mixtures	**140.01 (100.28–247.53)**	130.93 (115.21–160.92)	**175.89 (152.33–235.65)**	**134.06 (119.03–160.37)**	**113.55 (97.05–150.92)**
CARE/GSRUN	130.2 (106.21–173.89)	136.34 (115.58–181.64)	**171.01 (146.92–253.56)**	**133.75 (118.92–159.61)**	**111.62 (97.05–142.45)**

*f_k_*: Number of individuals captured *k* times. *S*: number of distinct individuals captured. *p_tr_*: daily threshold capture probability for which 95% of individuals are expected to be included in the reference population. *CV*: coefficient of variation (degree of heterogeneity). LP: Lincoln-Petersen; MLP: Multiple Lincoln-Petersen; MPE: Mean Petersen estimate; IntJK: Interpolated jackknife; ME: Moment estimator; SC1: Sample coverage 1; SC2: Sample coverage 2; EE: Estimating equation.

The 95%-confidence interval is shown in brackets. Estimations that cover the reference population size are highlighted in bold.

Even for the primary periods for which there was a tendency of overestimation or underestimation, the 95%-confidence interval of at least 50% of the estimators contained the reference population size (Table 2). In total, 29 out of 50 (58%) estimations without covariates included the reference population size. Of the ten estimates using covariates, seven included the reference population size.

The M_h_ model of Chao (ME) implemented in MARK/CAPTURE was the only model that always included the reference population size. The truncated geometric distribution overestimated the reference population size most strongly and included it only once despite a wide 95%-confidence interval.

MARK/CAPTURE selected different models as appropriate for different data sets: model M_t_ for November 1985, model M_th_ for January, March and November 1986, and the interpolated jackknife model M_h_ for January 1987. Except the first primary period, a heterogeneity model was chosen and three out of five models were heterogeneity and time dependent models.

Based on the AIC-value, the best models of the GSRUN model set of program CARE-2/GSRUN were model M_t_ for November 1985 and November 1986, model M_h_ including the covariate “hut” for January 1987, and model M_th_ including both covariates “age” and “hut” for the remaining primary periods (January 1986 and March 1986).

The ranking of the performance of the evaluated estimators is shown in Table 3 (for exact values see Table S1). Regarding relative bias, precision, and accuracy the top 1, 3, and 4 models, respectively, performed similarly (difference <50% of the best model; Table 3; Table S1). SC1, IntJK, ME, and EE belonged to these top models. For the cases which reflect the total population (sample periods 1 and 2), SC1 came closest to the true population. The truncated geometric distribution performed worst and LP, GSRUN, and the Finite mixtures model ranked among the lowest on all three criteria.

**Table 3:** Ranking of estimators for the partly independent data.

Rank	Relative bias	Relative precision	Relative accuracy	95%-Confidence interval width
1	**MPE**	**M_h_ EE**	**M_h_ ME**	**MLP**
2	M_h_ IntJK	**M_h_ SC2**	**M_h_ EE**	**LP**
3	MLP	M_h_ ME	**M_h_ SC1**	M_h_ MPE
4	M_h_ SC1	M_h_ SC1	**M_h_ IntJK**	M_h_ EE
5	M_h_ ME	M_h_ IntJK	Appropriate	M_h_ SC2
6	M_h_ EE	Appropriate	MLP	Appropriate
7	Appropriate	MLP	M_h_ SC2	GSRUN
8	M_h_ SC2	Finite mixtures	MPE	M_h_ IntJK
9	Finite mixtures	GSRUN	Finite mixtures	Finite mixtures
10	GSRUN	MPE	GSRUN	M_h_ SC1
11	LP	LP	LP	M_h_ ME
12	Tr. Geom. Distribution	Tr. Geom. Distribution	Tr. Geom. Distribution	Tr. Geom. Distribution

LP: Lincoln-Petersen. MLP: Multiple Lincoln-Petersen. MPE: Mean Petersen estimate. Int. JK: Interpolated jackknife. ME: Moment estimator. SC1: Sample coverage 1. SC2: Sample coverage 2. EE: Estimating equation. Tr. geom. distribution: Truncated geometric distribution.

**Ranking positions with difference <50% of the best model are shown in bold.**

### Fully independent data sets

**Figure 2:**
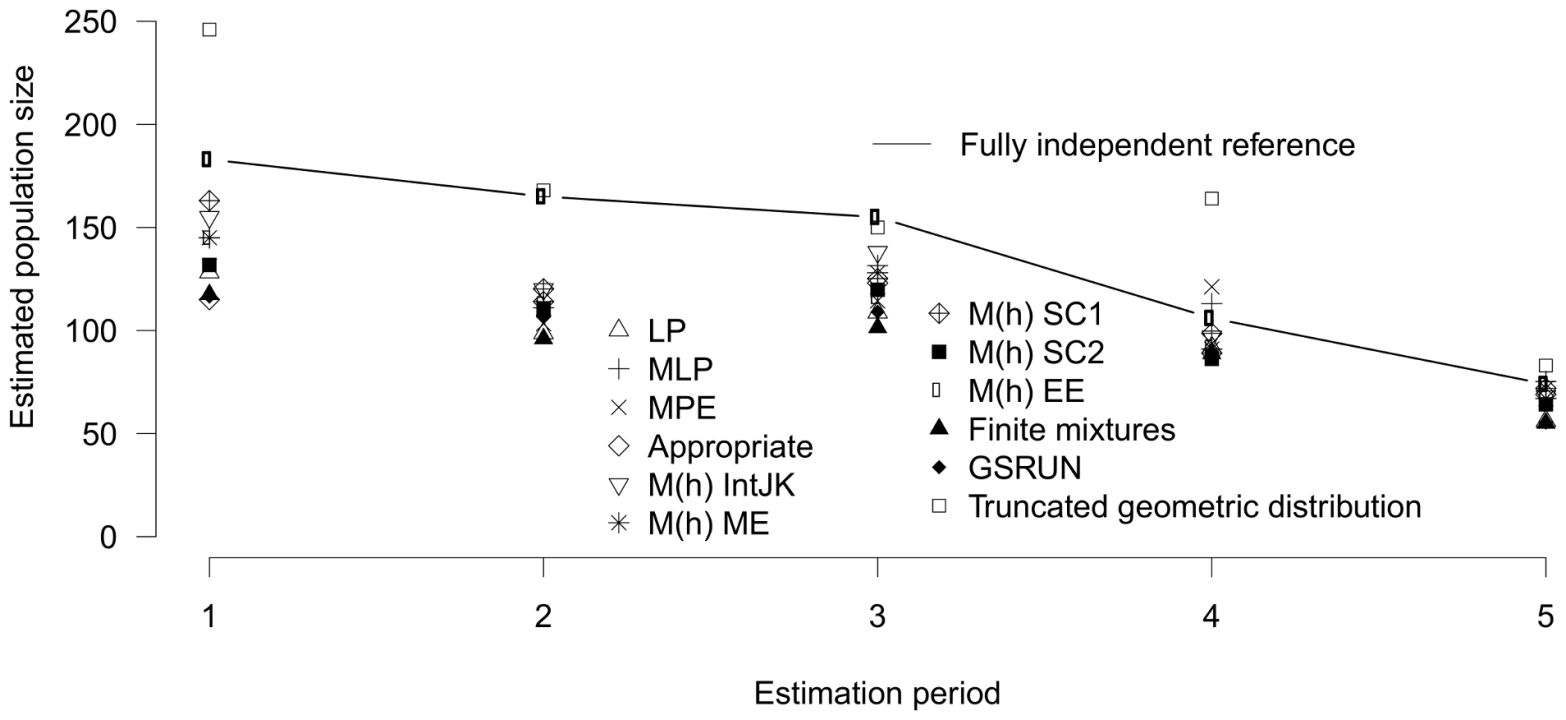
Population size estimates of fully independent entities. Comparison of different methods for population size estimates with the fully independent reference population sizes (connected by a line). LP: Lincoln-Petersen; MLP: Multiple Lincoln-Petersen; MPE: Mean Petersen estimate; IntJK: Interpolated jackknife; ME: Moment estimator; SC1: Sample coverage 1; SC2: Sample coverage 2; EE: Estimating equation.

For the fully independent data sets, all estimators, except the truncated geometric distribution, underestimated the reference population size in the first three primary periods, but were close to the reference population size in the remaining two periods (Fig. 2). In the first primary period, bias was moderate for SC1 and IntJK but the 95%-CI of the SC1 estimate was very broad. For the second period, surprisingly, all estimators performed poorly, except of the truncated geometric distribution. The truncated geometric distribution overestimated the reference population size three times and was twice very close to it. LP, SC2, the Finite mixtures model, and Care-2/GSRUN underestimated all reference population sizes strongly and their 95% CI covered the reference population size only once each (Table 4).

**Table 4:** Results for population size estimation with fully independent data.

Sample period	1985_11	1986_01	1986_03	1986_11	1987_01
Reference	183	165	155	106	74
*f_1_*	48	38	35	33	21
*f_2_*	15	23	19	26	14
*f_3_*	4	13	18	9	8
*f_4_*	1	3	15	2	5
*f_5_*	0	1	5	0	3
*f_6_*	0	2	3	0	0
*f_7_*	0	0	1	0	0
*f_8_*	0	0	0	0	0
S	68	80	96	70	51
p_tr_	0.092	0.122	0.171	0.259	0.451
CV	0.50	0.52	0.55	0.21	0.57
LP	128.23 (82.01–174.45)	98.75 (83.99–113.51)	108.77 (98.18–119.36)	**91.75 (74.14–109.36)**	56.11 (49.89**–**62.33)
MLP	144.91 (117.64–172.21)	114.03 (105.64–122.42)	131.52 (125.30–137.73)	**113.09 (102.67–123.50)**	**75.25 (70.47–80.03)**
MPE	131.19 (94.46–167.93)	103.27 (89.96–116.58)	114.35 (106.47–122.23)	**121.22 (86.78–155.66)**	**72.00 (64.89–79.12)**
MARK Appropriate	115 (93–156)	114 (96–151)	123 (110–150)	**89 (80–107)**	**69 (59–96)**
MARK M_h_ IntJK	**155 (124–205)**	120 (102–155)	**138 (118–178)**	**97 (85–121)**	**68 (59–90)**
MARK M_h_ ME	**145 (104–232)**	111 (95–149)	**128 (111–170)**	**91 (79–119)**	**67 (57–95)**
CARE M_h_ SC1	**163.0 (117.15–255.15)**	120.2 (99.81–152.76)	125.2 (112.21–145.04)	**98.6 (86.09–122.96)**	**71.9 (60.47–92.32)**
CARE M_h_ SC2	**131.8 (98.94–204.51)**	110.3 (93.63–137.48)	119.8 (107.22–137.58)	86.2 (76.85–103.10)	**64.2 (54.41–82.27)**
CARE M_h_ EE	**145 (107.46–215.22)**	113 (96.94–138.09)	116.2 (106.06–130.99)	**91.9 (81.79–107.69)**	**67.2 (57.92–82.05)**
Tr. Geometric Distribution	**246 (182–347)**	**168 (143–200)**	**150 (137–165)**	164 (135–204)	**83 (73–96)**
Finite Mixtures	117.51 (94.2–161.58)	96.06 (88.06–111.98)	101.43 (98.07–110.24)	**88.83 (79.38–107.82)**	55.12 (52.29–64.19)
CARE/GSRUN	116.47 (93.68–159.49)	106.88 (90.16–151.15)	109.05 (100.11–137.45)	**89.81 (80.08–108.93)**	55.33 (52.47–63.74)

*f_k_*: number of individuals captured *k* times. *S*: number of distinct individuals captured. *p_tr_*: daily threshold capture probability for which 95% of individuals are expected to be included in the reference population. *CV*: coefficient of variation (degree of heterogeneity). LP: Lincoln-Petersen; MLP: Multiple Lincoln-Petersen; MPE: Mean Petersen estimate; IntJK: Interpolated jackknife; ME: Moment estimator; SC1: Sample coverage 1; SC2: Sample coverage 2; EE: Estimating equation.

The 95%-confidence interval is shown in brackets. Estimations that cover the reference population size are highlighted in bold.

Capture intensity was high for the first two primary periods so that very few, if any, individuals may have been missed in our reference populations, even if individual capture probability was as low as 0.092 (Table 4). The reference populations of the last two primary periods, in contrast, did not include all individuals with low capture probability. Especially for the last primary period, daily threshold capture probability for which 95% of individuals are expected to be included in the reference population was 0.451. The coefficient of variation among individual capture probabilities was 0.5–0.57 except for primary period November 1986 (0.21).

Except for the second primary period, where just the estimate of the truncated geometric distribution included the reference population size, the 95%-confidence interval of two estimators (IntJK, ME) contained the reference population size in every primary period (Table 4). The truncated geometric distribution also covered the reference population size in four out of five cases. Altogether, 27 out of 50 (54%) estimators without covariates comprised the reference population size whereas just two out of ten estimates using covariates included the reference population size.

MARK/CAPTURE selected different models as appropriate: model M_t_ for November 1985, M_th_ for January 1986 and 1987 as well as March 1986 (for the last two no differences between M_th_ and M_h_ were detected) and M_0_ for November 1986. A heterogeneity model was chosen in three out of five primary periods. According to the AIC-values, the best models of the GSRUN model set in program CARE-2/GSRUN were model M_t_ for November 1985 and January 1987, model M_th_ with both covariates “age” and “hut” for January and March 1986, and model M_0_ for November 1986. Hence, in two out of five primary periods a heterogeneity model was chosen.

The ranking of the performance of the evaluated estimators for fully independent data sets is shown in Table 5 (for exact values see Table S1). MLP and SC1 performed best and second best, respectively, regarding all criteria except the width of the confidence interval, for which the latter performed comparably badly. IntJK and MPE followed with performance values similar to SC1 (Table 2, Table S1). Expectedly, LP did rank very low but surprisingly, the models using covariates obtained the lowest ranking positions.

**Table 5:** Ranking of estimators for the fully independent data.

Rank	Relative bias	Relative precision	Relative accuracy	95%-Confidence interval width
1	**MLP**	**MLP**	**MLP**	**MLP**
2	**M_h_ SC1**	M_h_ SC1	M_h_ SC1	**Finite Mixtures**
3	**M_h_ IntJK**	MPE	M_h_ IntJK	LP
4	**MPE**	M_h_ IntJK	MPE	GSRUN
5	M_h_ ME	Tr. Geom. Distribution	Tr. Geom. Distribution	MPE
6	M_h_ EE	M_h_ ME	M_h_ ME	Appropriate
7	Tr. Geom. Distribution	M_h_ EE	M_h_ EE	M_h_ EE
8	Appropriate	Appropriate	Appropriate	M_h_ SC2
9	M_h_ SC2	M_h_ SC2	M_h_ SC2	M_h_ IntJK
10	LP	LP	LP	M_h_ SC1
11	GSRUN	GSRUN	GSRUN	M_h_ ME
12	Finite Mixtures	Finite Mixtures	Finite Mixtures	Tr. Geom. Distribution

LP: Lincoln-Petersen. MLP: Multiple Lincoln-Petersen. MPE: Mean Petersen estimate. Int. JK: Interpolated jackknife. ME: Moment estimator. SC1: Sample coverage 1. SC2: Sample coverage 2. EE: Estimating equation. Tr. geom. distribution: Truncated geometric distribution.

Ranking positions with difference <50% of the best model are shown in bold.

MLP had the lowest width of the confidence interval, followed by the Finite mixtures model of Pledger (2000). However, the Finite mixtures performed worst for all other criteria.

## Discussion

The few studies that evaluated the performance of different estimators using data collected from populations of at least almost known size indicate that usually heterogeneity models perform better than models that ignore individual heterogeneity in capture probability [12], [13], [15], [18], [20]. Link (2003) anticipated that it may be very difficult to select among heterogeneity models because he expected that most will perform similarly well [50]. Our novel approach to create reference population sizes for evaluating the performance of population size estimators showed that the two best performing estimators resulted in rather similar estimates and confidence intervals but that there were considerable differences to other heterogeneity models for some reference populations. The assumption of individual heterogeneity was confirmed by a CV between 0.50 and 0.62 except for November datasets, which showed a CV between 0.21 and 0.50. A lower degree of heterogeneity in November each year might be caused by the absence of newly hatched juveniles [21].

Both approaches of creating reference populations resulted in similarities and differences in the overall pattern of performance of estimators. All estimators, except the truncated geometric distribution, underestimated the reference population size in the first two primary periods and were closer to it in the following primary periods. They overestimated the reference population size in the last periods only for the partially independent data set (Figs 1 & 2). These patterns can be explained by differences in the distribution of capture probabilities of the individuals in the biological entities. Since the first two reference populations were constructed based on more than 31 capture occasions, they likely included most individuals with near zero-capture probabilities. Larger numbers of individuals with low capture probabilities create particular challenges for population size estimators [28] and all estimators are assumed to underestimate population size in such cases [5]. This assumption is corroborated by our results except for the truncated geometric distribution. This may be explained by the geometric distribution entailing a long tail of low capture probabilities whereas the other methods do not or not sufficiently account for individuals with low capture probabilities (compare [51]). Capture experience suggested that the number of individuals with low capture probability was non-negligible in our study, though presumably not large, which may explain overestimation by the truncated geometric distribution.

In later primary periods, the reference population sizes include increasingly fewer individuals with low capture probability. This resulted in a better average performance of the estimators. In the last primary period, half of the estimators overestimated the partially independent reference population size whereas the remaining ones were close to it. Overestimation can be explained by the non-independence of the data used to construct the reference population and the data used to estimate its size. It reflects the different performance of the estimators when a large percentage of the individuals were caught but a considerable number was caught less than twice.

Whereas the relative performance of the evaluated estimators was rather inconsistent for the four performance criteria when tested with the partly independent data, it was highly consistent when testing with the fully independent data. For the latter, the multiple Lincoln-Petersen estimator performed best on all criteria except for coverage of the true value by the 95%-CI, followed by the first sample coverage (SC1) of Lee and Chao (1994) and the interpolated jackknife estimator (intJK) as implemented in CAPTURE and MARK. The comparably good performance of the multiple Lincoln-Petersen estimator may seem surprising since the basic Lincoln-Petersen estimator assumes equal capture probability during the two capture periods [3]. However, the pooling of occasions in our proposed multiple Lincoln-Petersen estimator (MLP) reduces heterogeneity and increases capture probability. This strategy accounted for daily individual capture heterogeneity comparable to the strategies of the M_h_ estimators as shown by similarities of the estimates (Table 4). Only for the first primary period, which likely included also individuals with near-zero capture probability, it was less efficient to account for heterogeneity than SC1 and the interpolated jackknife estimator. Therefore, MLP can be used as minimum population size as long as a sufficient number of individuals with high catchability are included.

The relative good performance of the jackknife estimator corroborates the conclusions of previous simulations that were based on virtually created distributions of capture probability [4], [11], [34], [52], [53], [54]. It was very close to the reference population size for the later primary periods in which heterogeneity was lower than for the first primary periods and capture rates were high, despite its tendency to overestimate for theoretically constructed datasets if heterogeneity is low [33] or capture rate very high [30]. This argues for caution when extrapolating from simulations with virtual data to real populations since the exact characteristics affecting the distribution of capture probabilities in wild populations will remain unclear. Here data from real populations can inform future simulations to construct virtual data that cover better distributions found in real populations.

The first sample coverage (SC1) resulted in very similar estimates and similar 95%-CIs as the interpolated jackknife [intJK]. Also, all performance criteria were very similar. Thus, as predicted by Link (2003) for all heterogeneity models, it is difficult to differentiate these two models and both may be used equally [50]. Notwithstanding, underestimation was less for SC1 for the first primary period with the highest number of individuals with low capture probability. Although SC1 is known to work well above a CV of 0.4 [29], [30], for our data the estimator worked well even when CV was smaller than 0.4 (Tables 2 and 4). However, a CV<0.4 combined with sparse data may lead to higher standard errors [29] and therefore wider 95%-CIs as shown in November 1985 (Tables 2 and 4). The same was the case for the interpolated Jackknife estimator.

Chao et al. (1992) and Lee & Chao (1994) indicated that it may be difficult to select between SC1 and SC2 [29], [30]. For our data SC1 covered the reference population more often than SC2 and its accuracy was considerably higher than that of SC2 (Table S1). Its tendency to underestimate was much stronger than that of SC1, for the partially independent data set even stronger than that of the moment estimator (ME).

For ME, most performance criteria were similar to those of the interpolated jackknife and SC1 (Table S1). It covered the reference population more often for the partly independent data set than the interpolated jackknife showing at the same time the highest accuracy among all estimators. However, it performed slightly less good than the interpolated jackknife and SC1 in terms of precision for both data sets (Table S1). In terms of the width of the confidence interval, it ranked lowest and second lowest of all estimators (Table 5).

Chao (1988, 1989) suggested that the moment estimator should work comparably well, if many individuals are captured just once or twice (low overall capture probability), as it is based on *f_1_* and *f_2_* (i.e. individuals captured once or twice) while the interpolated jackknife estimator should work best when many individuals are captured more than twice because it uses a linear combination of all capture frequencies [36], [37]. However, for our data set with the fewest individuals captured more than twice (November 1985), it underestimated the reference population more than the interpolated jackknife and considerably more than SC1. Rather, our results support the idea that ME is negatively biased and can be seen as lower bound estimator in the presence of capture heterogeneity [36], [37]. Furthermore, smaller population size that reduces the capture frequencies as in our fully independent dataset leads to an increased standard error of the ME [36] resulting in a very large 95%-CI.

The estimating equation [EE] requires a large number of capture-recapture data to obtain reliable estimates of time, individual heterogeneity, and behaviour effects [11]. This clearly explains the better performance in partly independent in comparison to fully independent data (Tables 2 and 4). For this data set it showed a very good accuracy and a small confidence interval.

The poorest relative performance was exhibited by the Finite mixtures model of Pledger (2000), followed by GSRUN [25], Chapman's Lincoln-Petersen estimate [LP] [3], [4], the second sample coverage of Lee and Chao (1994), and the model selected as appropriate by CAPTURE. The relative poor performance of the latter confirms that the model selection procedure of CAPTURE does not work satisfactorily [4], [9], [11], [53]; Stanley & Burnham (1998) even stated that this procedure in CAPTURE selects an inappropriate model [55].

While the poor performance of the LP estimate was expected because of its assumption of equal capture probability, we were surprised that GSRUN and the Finite mixtures model showed a rather poor performance. Not only did they tend to strongly underestimate, their 95%-CI included only once the reference population size for the fully independent reference populations. For the partly independent reference populations, they also did not perform well. The relative poor performance of GSRUN might be explained by having selected the wrong covariates. However, we selected covariates that, based on AIC values and direct observations, likely explain part of the heterogeneity observed in capture probability. Huts with their differences in structure as an expected explanatory variable were included only for a few datasets in the best models (based on AIC) but did not improve the performance of the estimators. This may be explained by our capture experience showing that the preferred position individuals occupied at the huts influenced the chance of capturing them. This factor varied more within than across huts and is difficult to model as covariate but likely had a stronger effect than the covariates we could measure. Fitting models with covariates under such conditions remains challenging [56].

Presence of individuals with low capture probabilities and absence of a structure that allows clear groupings of capture probabilities in finite groups may also be the reason why the mixture model of Pledger (2005) [28] performed on average relatively poorly. In line with this explanation, it was among the best for the partially independent data sets in which most or all of the individuals with low capture probability were removed. Pledger's (2005) model also performed less well than the appropriate model in CAPTURE in a study of the giant day gecko (*Phelsuma madagascariencsis grandis*) population of known size released in the Masoala rainforest exhibit (Zurich Zoo) [12].

With the advent of a range of estimation methods that model temporal, behavioural, and individual variability of capture probabilities, the estimation of population size by fitting recapture frequencies to mathematical distributions has fallen into disfavour. Notwithstanding, a truncated geometric distribution may result from modelling the capture process, e.g., if average capture rate is proportional to home range area [3]. Also, recently Nitwitpong et al. (2013) suggested based on theoretical and simulation results that the truncated geometric distribution should approximate capture frequencies well, and better than other distributions, when there is a long tail of low capture probabilities [51]. Our results showed that for such reference populations, it was the only method that did not underestimate the reference population size. While the method may be recommended for such data, it did overestimate the reference population substantially for several other reference populations, especially for the partly independent data set. A further disadvantage was the worst performance in terms of confidence interval width. To better understand under which conditions the truncated geometric distribution may be used appropriately and avoid underestimation, we suggest further simulations for data with a long tail of low capture probabilities and applications to populations of known size for which it is also known that many individuals have low capture probability.

## Conclusion

Selecting the most appropriate population size estimator and obtaining reliable estimates requires sufficient capture information. There is no single estimator that performs best and results in very good estimates for all data sets. If individual heterogeneity is high (CV>0.4) either the interpolated jackknife [33] as implemented in CAPTURE/MARK or SC1 [29] may be selected, both performing very similarly and adequately for most of our data sets. Only for the first primary period, which likely included the largest percentage of individuals with near-zero capture probability, was its bias clearly less. As the first primary period corresponds to the complete real population (partially independent data) or the real number of individuals surviving from the first to later primary periods (fully independent data), SC1 may be preferable for populations similar to the geckos in this study, unless the wide 95%-CI is of more concern than bias. If few individuals with low capture probabilities are present, the moment estimator [35] implemented in CAPTURE/MARK may be a better choice.

If in contrast underestimation is of concern, e.g. when assessing the impact of an invasive species, and if it is expected that a considerable number of individuals have low capture probability, the truncated geometric distribution may be the best choice when used together with the moment estimator to also get an estimate of the lower bound of population size. If a large number of capture occasions can be pooled and the number of individuals with very low capture probability is likely limited, our new multiple Lincoln-Petersen estimate may be a strategy that deals with heterogeneity as good to modelling individual capture heterogeneity; however, further tests with populations of known size and simulation studies are needed to corroborate this conclusion.

To improve the robustness of guidelines for the selection of suitable estimators for field data, we recommend similar studies for other species as the distribution of their capture probabilities may deviate from the geckos in our study. Capture frequency distributions from real populations may also profitably be used to construct virtual capture data for simulation studies that realistically reflect the variability of capture probabilities in real populations.

## Supporting Information

Table S1 **Ranking values of each estimator calculated for the first five periods.**
(DOCX)
