# Supplementary material for: Reliability of Different Mark-Recapture Methods for Population Size Estimation Tested against Reference Population Sizes Constructed from Field Data
Source: PLoS One. 2014 Jun 4;9(6):e98840. doi: 10.1371/journal.pone.0098840 (PMC4045897; doi:10.1371/journal.pone.0098840)
Supplement: Table S1 — Ranking values of each estimator calculated for the first five periods. (DOCX) [file pone.0098840.s001.docx]

# Supporting Information

S1: Ranking values of each estimator calculated for the first five periods.

| **Estimator** | **partly independent analyses** | | | **fully independent analyses** | | |
| --- | --- | --- | --- | --- | --- | --- |
|  | **relative bias** | **relative precision** | **relative accuracy** | **relative bias** | **relative precision** | **relative accuracy** |
| LP | -0.1633 | -0.0213 | 0.0479 | -0.2751 | -0.0738 | 0.1494 |
| MLP | 0.0370 | 0.0099 | 0.0112 | -0.1170 | -0.0088 | 0.0224 |
| MPE | -0.0175 | 0.0172 | 0.0175 | -0.1606 | -0.0167 | 0.0425 |
| MARK Appropriate | -0.0528 | 0.0071 | 0.0099 | -0.2230 | -0.0468 | 0.0966 |
| MARK M_h_ IntJK | 0.0338 | 0.0067 | 0.0078 | -0.1403 | -0.0184 | 0.0381 |
| MARK M_h_ ME | -0.0449 | 0.0037 | 0.0057 | -0.1890 | -0.0342 | 0.0699 |
| CARE M_h_ SC1 | 0.0418 | 0.0055 | 0.0072 | -0.1343 | -0.0161 | 0.0341 |
| CARE M_h_ SC2 | -0.0930 | -0.0035 | 0.0121 | -0.2315 | -0.0524 | 0.1060 |
| CARE M_h_EE | -0.0503 | 0.0031 | 0.0057 | -0.1996 | -0.0382 | 0.0781 |
| Tr. Geometric Distribution | 0.5882 | -0.2960 | 0.6419 | 0.1998 | -0.0282 | 0.0681 |
| Finite Mixtures | -0.1222 | -0.0100 | 0.0250 | -0.3077 | -0.0927 | 0.1873 |
| CARE/GSRUN | -0.1355 | -0.0134 | 0.0317 | -0.2835 | -0.0789 | 0.1592 |

LP: Lincoln-Petersen; MLP: Multiple Lincoln-Petersen; MPE: Mean Petersen estimate; IntJK: Interpolated jackknife; ME: Moment estimator; SC1: Sample coverage 1; SC2: Sample coverage 2; EE: Estimating equation.
